# Diagnostik der periprothetischen Infektion: Entwicklung eines evidenzbasierten Algorithmus der Arbeitsgruppe implantatassoziierte Infektion der Arbeitsgemeinschaft Endoprothetik

**DOI:** 10.1007/s00132-020-03940-6

**Published:** 2020-07-14

**Authors:** H. Mühlhofer, N. Renz, A. Zahar, M. Lüdemann, M. Rudert, R. Hube, L. Frommelt, R. Ascherl, C. Perka, R. von Eisenhart-Rothe

**Affiliations:** 1 Klinik und Poliklinik für Orthopädie und Sportorthopädie, Klinikum rechts der Isar, Technische Universität München, Ismaninger Str. 22, 81675 München, Deutschland; 2 Centrum für Muskuloskeletale Chirurgie, Campus Charité Mitte, Charite, Luisenstraße 64, 10117 Berlin, Deutschland; 3 Helios Klinikum Emil von Behring, Walterhöferstr. 11, 14165 Berlin, Deutschland; 4 Orthopädische Klinik, König-Ludwig-Haus, Brettreichstraße 11, 97074 Würzburg, Deutschland; 5 Orthopädische Chirurgie München, OCM Klinik München, Steinerstr. 6, 81369 München, Deutschland; 6 Helios ENDO-Klinik Hamburg, Holstenstraße 2, 22767 Hamburg, Deutschland; 7 Kirchham, Deutschland

**Keywords:** Biofilm, Gelenkprothese, Protheseninfektionen, Sensitivität und Spezifität, Chirurgische Diagnosetechniken, Biofilm, Joint prosthesis, Prosthesis-related infections, Sensitivity and specificity, Surgical diagnostic technic

## Abstract

**Hintergrund:**

Die Behandlung periprothetischer Infektionen (PPI) ist eine der größten Herausforderungen im Bereich der Endoprothetik. Der möglichst sichere Ausschluss oder die Bestätigung einer periprothetischen Infektion ist die Voraussetzung für jede Revisionsoperation und kann für den behandelnden Orthopäden und Unfallchirurgen eine große Herausforderung darstellen. Eine sichere evidenzbasierte präoperative Diagnostik ist im Sinne des Patienten notwendig, um einerseits eine periprothetische Infektion zu erkennen sowie die entsprechende chirurgische und antibiotische Therapie zu planen und andererseits unnötige zweizeitige Wechsel zu vermeiden.

**Ziel der Arbeit:**

Ziel ist es, ein evidenzbasiertes problem- und prioritätenbasiertes Vorgehen zu entwickeln und dies in einem transparenten und standardisierten Algorithmus zusammenzufassen.

**Methode:**

Durch systematische Literaturrecherche wurden relevante Arbeiten identifiziert und im Rahmen von Expertenrunden bewertet. Nach Extraktion der Daten erfolgte die Berechnung von Sensitivität, Spezifität, positiver und negativer Likelihood-Ratio sowie positiver und negativer prädiktiver Werte. Im Rahmen von 4 Treffen wurden die entsprechenden Studien der Arbeitsgruppe für implantatassoziierte Infektionen präsentiert und analog zu Standard-Delphi-Runden durch die einzelnen Experten bearbeitet und bewertet. Gemäß der Prioritätenliste der Expertenrunde erfolgte die Entwicklung eines zur ISO (International Organization for Standardisation) konformen Algorithmus.

**Ergebnisse:**

Der entwickelte Algorithmus ist eine Abfolge von evidenzbasierten Prozessen gemäß der verwendeten ISO-Norm. Gemäß der durch die Expertenrunde priorisierten Haupt- und Nebenkriterien erfolgte die Entwicklung logisch strukturiert und problemorientiert.

**Schlussfolgerung:**

Der Ausschluss einer periprothetischen Infektion ist von enormer Bedeutung vor einer Revisionsoperation und entscheidet in vielen Fällen über den Erfolg und die Invasivität der Operation. Die Diagnose „periprothetische Infektion“ erfordert eine substanzielle Veränderung der therapeutischen Strategie. Der durch die Arbeitsgruppe entwickelte Algorithmus fasst Positionen aus der aktuellen Literatur und spezielle Expertenmeinungen zusammen, dies ermöglicht einen transparenten diagnostischen Ansatz im Sinne einer Standard Operation Procedure.

## Abstract (en)

**Background:**

Prosthetic joint infections (PPI) will challenge orthopaedic surgeons and the health care system in the coming years. Evidence-based and reliable preoperative diagnostics are necessary for success in the field of revision arthroplasty. Especially the preoperative detection of PPI is important with respect to the treatment strategy.

**Aim:**

The aim of this study was to develop a detailed and structured standard operating procedure (SOP) to detect PPI preoperatively.

**Methods:**

A systematic literature research was performed and relevant articles identified. After extracting the data, statistical calculations of sensitivity, specificity, positive/negative predictive value and positive/negative likelihood ratio were performed. The results were discussed and evaluated in four meetings analogously to standard Delphi rounds by the workgroup of implant-associated infections of the German AE (Arbeitsgemeinschaft Endoprothetik). An algorithm for the diagnostic approach according to ISO 5807 was made.

**Results:**

The standardized algorithm combines a sequence of evidence-based procedures with detailed and structured main and additional criteria to every critical step in the diagnostic approach.

**Conclusion:**

The detection of PPI is of tremendous importance prior to revision arthroplasty and determines its success or failure. The diagnosis “prosthetic joint infection” requires a substantial change with respect to treatment concepts. The algorithm summarizes current literature and specialized expert opinions in a modern standardized format for a transparent diagnostic approach.

## Einleitung

Die periprothetische Infektion (PPI) einer Endoprothese stellt eine der schwerwiegendsten Komplikationen im Bereich der Orthopädie dar und ist eine der Hauptursachen von Revisionsoperationen. Aufgrund der demographischen Veränderung unserer Gesellschaft mit einem zunehmenden Anteil älterer Menschen mit hohem bis sehr hohem Funktionsanspruch wird die Zahl der implantierten Endoprothesen in den nächsten Dekaden deutlich zunehmen. Schätzungen aus den USA gehen von einem erwarteten Anstieg von 600 % der implantierten Kniegelenksendoprothesen bis ins Jahr 2030 aus. Implantationen von Hüftgelenksendoprothesen werden sich in diesem Zeitraum verdreifachen, was unweigerlich zu einer signifikanten Zunahme der Revisionsoperationen führen wird. [41]. Studien beziffern die Inzidenz der PPI nach Primärimplantationen auf ca. 0,2–1,1 %. Bei Revisionsoperationen kann diese bis zu 5 % betragen [81].

Grundsätzlich können periprothetische Infektionen in akute Infektionen und Low-Grade-Infektionen eingeteilt werden. Akute Infektionen werden durch hochvirulente Erreger, wie z. B. Staphylococcus aureus*, *hervorgerufen und treten entweder postoperativ durch direkte Kolonisation oder durch eine hämatogene Streuung auf. Sie sind oft durch klare Infektionszeichen wie Fieber und erhöhte laborchemische Entzündungsparameter gekennzeichnet. Als potenzieller Fokus für eine hämatogene Streuung kommen alle Arten von bakteriellen Infektionen im Körper infrage. Als klassische Vertreter sind hier kardiovaskuläre Infektionen (Endokarditis, Schrittmacherinfektion, Katheterinfektionen) bzw. Infektionen im Hals-Nasen-Ohren-Bereich, im Bereich der Zähne oder im Urogenitaltrakt zu nennen [67]. Low-Grade-Infektionen sind chronische Infekte, welche klinisch durch Prothesenlockerung und unspezifische Schmerzen im Bereich der Endoprothese bei Abwesenheit von akuten Entzündungszeichen auffallen. Diese werden oftmals durch niedrig virulente Erreger wie z. B. Staphylococcus epidermidis oder Cutibacterium (früher: Propionibacterium*) *acnes hervorgerufen [72]. Low-Grade-Infektionen werden am ehesten durch eine Kolonisation der Implantatkomponenten im Rahmen der Erstimplantation verursacht.

Der möglichst sichere Ausschluss oder die Bestätigung einer periprothetischen Infektion ist die Voraussetzung für jede Revisionsoperation und kann für den behandelnden Orthopäden und Unfallchirurgen eine große Herausforderung darstellen [89]. Eine sichere evidenzbasierte präoperative Diagnostik ist im Sinne des Patienten notwendig, um einerseits eine periprothetische Infektion zu erkennen sowie eine entsprechende chirurgische und antibiotische Therapie zu planen und andererseits, um unnötige zweizeitige Wechsel zu vermeiden. Gerade die Diagnostik von Low-Grade-Infektionen kann jedoch sehr herausfordernd sein. Neben der Anamnese und der klinischen Untersuchung stellen Röntgenaufnahmen, laborchemische Entzündungsparameter, Gelenkaspiration zur Bestimmung von Zellzahl und Zelldifferenzierung sowie mikrobiologischer Untersuchung der Synovialflüssigkeit bewährte diagnostische Routinen dar [87].

In den letzten Jahren stehen neu entwickelte Testverfahren, wie die molekularbiologische Diagnostik von Biomarkern (z. B. Alpha-Defensin), antimikrobiellen Peptiden (AMP) und der Leukozytenesterasetest zur Synovia-Diagnostik, sowie molekulargenetische Untersuchungen (16S/28S-PCR, Multiplex-PCR) zur Erregerbestimmung aus mikrobiologischen Biopsien zur Verfügung. Der Stellenwert dieser diagnostischen Möglichkeiten ist oftmals unklar und der behandelnde Orthopäde steht vor der großen Herausforderung, unter Berücksichtigung der zur Verfügung stehenden finanziellen Ressourcen des Gesundheitssystems die adäquate präoperative Diagnostik zu wählen.

Aus diesem Grund hat die Arbeitsgruppe für implantatassoziierte Infektionen ein evidenzbasiertes problem- und prioritätenbasiertes Vorgehen entwickelt und dies in einem transparenten und standardisierten Algorithmus zusammengefasst.

## Material und Methoden

**Figure 1:**
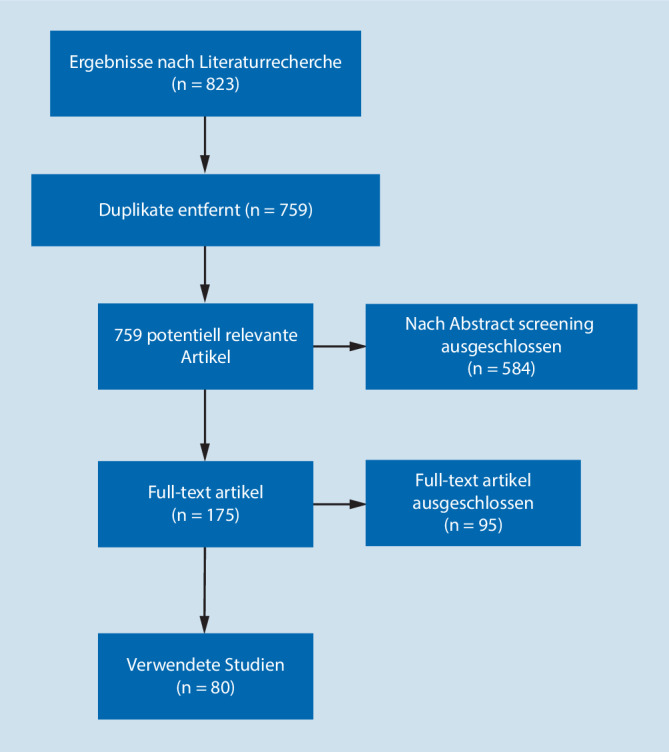

Im Rahmen der Treffen der Arbeitsgruppe für implantatassoziierte Infektionen der Arbeitsgemeinschaft Endoprothetik erfolgte eine systematische Literaturrecherche in Medline, Google Scholar und Web of Science mit folgenden Schlagworten (Frage_ AND/OR): prosthetic joint infection, implant-associated infection, biofilm, diagnosis, sonication, antibiotic treatment, microcalorimetry, Staphylococcus aureus, coagulase-negative staphylococci, Propionibacterium, cutibacterium, rifampicin, implant retention, PCR, Maldi-TOF, serology, synovial fluid, C‑reactive protein level, THA, TKA, leukocyte esterase test, alpha-defensin test. Alle relevanten Publikationen wurden hinsichtlich ihrer Methodik gemäß den Quadas- und Prisma-Kriterien geprüft, bevor sie berücksichtigt wurden [51]. Alle eingeschlossenen Studien wurden gemäß den EAST-Kriterien bewertet und eingeteilt [28–34]. Insgesamt konnten 823 Publikationen identifiziert werden, die unserer Suchkriterien entsprochen haben (Abb. 1). Weiterhin wurden vor allem Studien berücksichtigt, die eine international gängige Definitionen einer periprothetischen Infektion wie MSIS [59] (Musculoskeletal Infection Society) [76], IDSA (Infectious Diseases Society of America) [58], International Consensus Meeting [61] oder EBJIS (European Bone and Joint Infection Society) [68] angewandt haben. Insgesamt konnten so 80 Studien herangezogen werden. Aus diesen Studien wurden die entsprechenden Daten extrahiert und folgende statistische Tests berechnet, falls dies noch nicht in der Originalpublikation erfolgt ist: Sensitivität, Spezifität, positive und negative Likelihood-Ratio, positive und negative prädiktive Werte.

Im Rahmen von 4 Treffen wurden die entsprechenden Studien der Arbeitsgruppe für implantatassoziierte Infektionen präsentiert und analog zu Standard-Delphi-Runden durch die einzelnen Experten bearbeitet und bewertet [69]. Gemäß der Prioritätenliste der Expertenrunde erfolgte die Entwicklung eines zur ISO (International Organization for Standardisation) konformen Algorithmus. Zur Anwendung kam die ISO-Norm 5807 (Informationsverarbeitung) modifiziert nach ITU‑I, welche ursprünglich Dokumentationssymbole für Programm und Systemabläufe von Telekommunikationsprogrammnetzen bereitstellt. Durch die Modifikation erfolgte die Integration von Datenfluss-Flussdiagrammen (wie Algorithmen). So wird eine logische und standardisierte Entscheidungsfindung ermöglicht [39]. Hierzu werden verschiedenen Eingangs- und Ausgangskriterien definiert, die von Prozess- und Entscheidungshexagons unterschieden werden. Durch Verwendung von Checklisten konnten die Entscheidungssymbole auf ein Minimum beschränkt werden. Grundsätzlich sollte eine DIN-A 4-Seite nicht überschritten werden.

## Ergebnisse der Arbeitsgruppe

**Figure 2:**
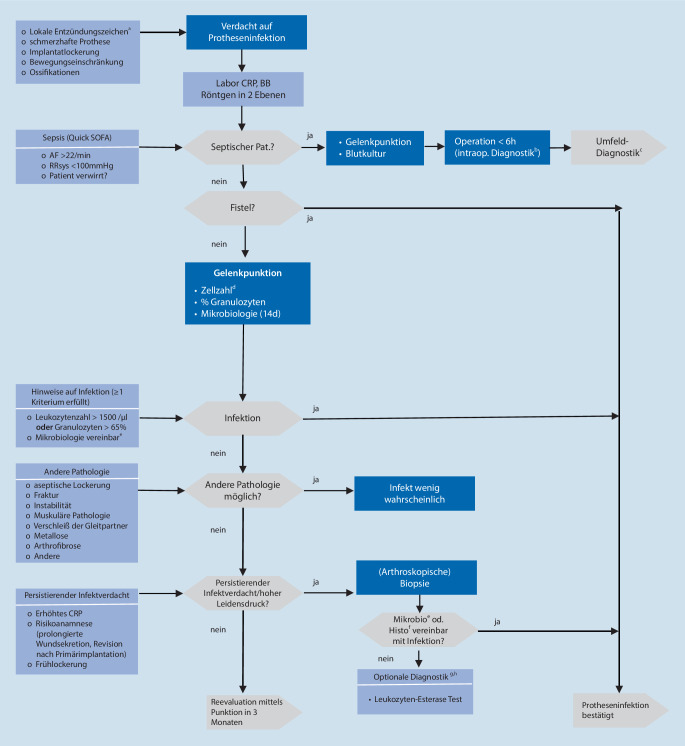

Der entwickelte Algorithmus ist eine Abfolge von evidenzbasierten Prozessen gemäß der verwendeten ISO-Norm. Gemäß der durch die Expertenrunde priorisierten Haupt- und Nebenkriterien erfolgte die Entwicklung logisch strukturiert und problemorientiert. Im horizontalen Flow werden Nebenkriterien dargestellt, im vertikalen Flow die Hauptkriterien. Checklisten wurden am linken Rand positioniert ([39]; Abb. 2).

## Eingangskriterium

Als Eingangskriterium, also Startpunkt des Algorithmus wurde von der Expertenrunde der „V. a. eine periprothetische Infektion“ angenommen. Insgesamt wurde 39 Studien mit 312.946 Patienten zu diesem Thema eingeschlossen.

Große Multizenterstudien konnten Risikofaktoren bzw. Patientengruppen mit erhöhtem Risiko einer periprothetischen Infektion identifizieren. Gerade die Lockerung der Implantatkomponenten, periartikuläre Ossifikationen, eine Bewegungseinschränkung des Gelenkes sowie Schmerzen im Bereich der Endoprothese und lokale Entzündungszeichen sollten den Verdacht auf einen periprothetischen Low-Grade-Infekt lenken [43]. Die Risikofaktoren und Evidenzgrade der Studien sind ausführlich in Tab. 1 dargestellt.

**Table 1:** 

Risikofaktor	Jahr	Autor	LoE	(*n* = x)
Operationsdauer	2007	Huotari et al. [35]	LoE II	*n* = 8201
	2004	Småbrekke et al. [75]	LoE II	*n* = 31.750
	2010	Kurtz et al. [42]	LoE II	*n* = 69.663
	2009	Uçkay et al. [82]	LoE I	*n* = 6001
	1998	Berbari et al. [7]	LoE II	*n* = 26.505
Übergewicht	2009	Dowsey et al. [20]	LoE III	*n* = 1214
	2001	Peersman et al. [63]	LoE III	*n* = 6120
	2007	Lübbeke et al. [45]	LoE II	*n* = 2495
	2008	Dowsey et al. [21]	LoE III	*n* = 1207
	2008	Pulido et al. [66]	LoE III	*n* = 9245
	2012	Namba et al. [56]	LoE II	*n* = 30.491
	2013	Namba et al. [57]	LoE II	*n* = 56.216
	2009	Malinzak et al. [48]	LoE III	*n* = 8494
	2011	Peel et al. [62]	LoE III	*n* = 1200
Mangelernährung	2012	Berbari et al. [8]	LoE III	*n* = 678
Diabetes	2013	Namba et al. [57]	LoE II	*n* = 56.216
	2009	Malinzak et al. [48]	LoE III	*n* = 8494
	2001	Peersman et al. [63]	LoE III	*n* = 6120
	2011	Mraovic et al. [52]	LoE III	*n* = 1948
Immunsuppression	2008	Dowsey et al. [20]	LoE III	*n* = 1207
	2001	Peersman et al. [63]	LoE III	*n* = 6120
	2009	Jämsen et al. [36]	LoE II	*n* = 43.149
	2008	Pulido et al. [66]	LoE II	*n* = 9245
	2011	Peel et al. [62]	LoE III	*n* = 63
	1998	Berbari et al. [7]	LoE II	*n* = 26.505
	2008	Bongartz et al. [10]	LoE III	*n* = 462
Vorausgegangener Infekt	2009	Jämsen et al. [36]	LoE II	*n* = 43.149
Bakteriämie	2010		LoE III	*n* = 126
	2013	Coelho-Prabhu et al. [13]	LoE III	*n* = 678
	2001	Murdoch et al. [54]	LoE III	*n* = 80
Infektion weiterer Prothesen	1991	Murray et al. [55]	Level III	*n* = 159
	1996	Luessenhop et al. [46]	Level III	*n* = 145
Implantatlockerung	2013	Portillo et al. [65]	LoE I	*n* = 116
Bakteriurie	2013	Sousa et al. [77]	LoE III	*n* = 2497
Zahnstatus	2010	Berbari et al. [6]	Level III	*n* = 678
Schmerzhafte Prothese	2002	Virolainen et al. [83]	Level III	*n* = 68
Bewegungseinschränkung	2013	Parvizi et al. [61]	Level III	–
Ossifikationen	2002	Breitenseher et al. [12]	Level III	–
Lokale Entzündungszeichen	2000	Teller et al. [79]	Level III	*n* = 166

Es kann nicht empfohlen werden, bei jedem Patienten der zu einer Wechseloperation ansteht (z. B. Inlayverschleiß) unkritisch und ohne Risikoabwägung eine Gelenkpunktion durchzuführen. Einerseits darf man potenzielle Komplikation, wie z. B. iatrogene Infektionen, nicht unberücksichtigt lassen. Murray et al. zeigen in einer Level-I-Studie bis zu 5,1 % Komplikationen nach Hüftpunktionen (Nachblutungen, Hämatome, Nervenläsionen, Infektionen). Barrack et al. beschreiben eine deutlich niedrigere Komplikationsrate von 1 % [5]. Gemäß des Konsens der Expertengruppe stellt die Komplikationsrate durch die Punktion selbst nicht das vordergründige Problem dar. Die hohe Anzahl an Komplikationen in der Literatur kann so nicht nachvollzogen werden. Andererseits stellt ein weitaus größeres Problem die hohe Zahl an falsch positiven Befunden in der mikrobiologischen Kultur dar, die bei einer flächendeckenden Synovialpunktion auftreten und nicht unerhebliche klinische und juristische Konsequenzen mit sich bringen würden. Fehring et al. beschreiben eine Rate von 10,8 % falsch positiver mikrobiologischer Kulturen, Ali et al. eine von 7 %. Um eine Kontamination von einem Nachweis eines Pathogens unterscheiden zu können, soll die Zellzahl in der Synovialflüssigkeit zu Hilfe genommen werden; erfolgt ein Erregernachweis bei völlig normaler Zellzahl, ist dieses Resultat mit Vorsicht zu interpretieren und eher als Kontamination zu werten. Im Hinblick auf die Synoviaaspiration bei liegender H‑TEP muss auf die relativ hohe Anzahl an erfolglosen Punktionen (sogenannte Punctio sicca) hingewiesen werden. Diese treten überdurchschnittlich häufig bei aseptischen Hüften auf. Die Anzahl der falsch positiven Kulturen erhöht sich bei Punctio sicca von 7 auf 16,7 % [1] und die positive Likelihood-Ratio fällt von 8,9 auf 4,7. In diesem Zusammenhang muss darauf hingewiesen werden, dass auf eine Instillation mit Kochsalzlösung bei Punctio sicca verzichtet werden sollte.

### Fazit Punktion

Bei Vorliegen von Risikofaktoren für eine periprothetische Infektion (Tab. 1; Abb. 2) wird eine Punktion bzw. weitere Diagnostik empfohlen. Eine generelle, unkritische Punktion vor jeder Revisionsoperation kann nicht befürwortet werden.

## Checkliste Sepsis

Als einer der ersten Schritte muss, um das zeitliche operative Vorgehen und die Dringlichkeit zu planen, eine Sepsis oder ein septischer Schock ausgeschlossen werden. Dies ist vor allem bei hochakuten periprothetischen Infektionen, welche oft durch eine hämatogene Streuung entstehen, relevant. Wir haben hierzu 10 Studien eingeschlossen, die sich mit dem Thema einer periprothetischen Infektion und Sepsis auseinandersetzen: [18, 44, 74]. Die Verwendung der SIRS-Kriterien wurde gegenüber der moderneren SOFA-Kriterien diskutiert. Als Konsensus erfolgte die Festlegung auf Quick-SOFA bei gleicher Evidenzklasse aufgrund der einfacheren Anwendung. Vor dem Beginn einer kalkulierten Antibiotikatherapie sollte eine Synoviaaspiration des betroffenen Gelenks erfolgen, um durch eine weitere mikrobiologische Untersuchung des Punktats den verursachenden Erreger zu identifizieren (Empfehlung Klasse III) Die Entnahme von Blutkulturen ist zwingend notwendig. Gemäß einer Klasse-II-Empfehlung senkt die zeitnahe chirurgische Versorgung die Mortalität.

### Fazit Sepsis

Aufgrund der hohen Mortalitätsrate besteht bei V. a. auf ein septisches Krankheitsbild umgehender interdisziplinärer Handlungsbedarf mit Entnahme von Blutkulturen, zeitnaher Antibiotikatherapie und chirurgischer Intervention im Sinne einer „source control“.

## Laborchemische Entzündungsparameter

Im Rahmen der vielzitieren AAOS-Richtlinie wird zum Ausschluss einer periprothetischen Infektion ein kompliziertes Zusammenspiel von laborchemischen Parametern (Blutsenkungsgeschwindigkeit/C-reaktives Protein) verwendet [60]. In dieser Metaanalyse werden Level-I- und Level-II-Studien berücksichtigt, die errechneten Sensitivitäten variieren von 81–93 % bezüglich der BSG sowie von 73–95 % für das C‑reaktive Protein. Eine kürzlich veröffentlichte Metaanalyse von Berbari et al. zeigte ähnliche Ergebnisse. Bei einem Studienkollektiv von insgesamt 3909 Patienten errechnete sich eine gepoolte Sensitivität von 75 % (BSG) und 88 % (CRP) [6]. Hier muss jedoch berücksichtigt werden, dass die Studien mit den verwendeten Diagnosekriterien mehrheitlich deutliche Infektionen eingeschlossen haben und Low-Grade-Infektionen – welche typischerweise tiefe systemische Entzündungswerte hervorrufen – mit diesen Kriterien größtenteils nicht erfasst wurden. Somit sind die Sensitivitäten vermutlich deutlich überschätzt. Insbesondere beim CRP zeigen sich unbefriedigende Werte im Bereich der Sensitivität. McArthur et al. bestätigen diese Ergebnisse in ihrem Kollektiv mit einer relevant großen Gruppe von Patienten mit einem periprothetischen Infekt ohne relevante serologische Entzündungszeichen [50]. Im Gegensatz hierzu empfehlen die AAOS-Guidelines eine Synoviaaspiration nur bei erhöhten Entzündungszeichen im Blut. Dieses Vorgehen würde die periprothetischen Infektionen ohne erhöhte systemischen Entzündungswerte von einer weiteren invasiven Diagnostik ausschließen. Die entsprechenden Studien sind in Tab. 2 dargestellt. Die Arbeitsgruppe „implantatassoziierte Infektionen“ der Arbeitsgemeinschaft Endoprothetik hat in ihren Sitzungen die einzelnen Studien im Hinblick auf High- und Low-Grade-Infekte bewertet. Diese Bewertung lässt den Schluss zu, dass, obwohl in den AAOS-Richtlinien Level-I-Studien als Basis der Empfehlung verwendet werden, in diesen zum Teil relativ alten Studien die Zahl der Low-Grade-Infekte sehr gering beziehungsweise teilweise nicht vorhanden ist. Gerade Low-Grade-Infekte sind durch Abwesenheit von lokalen und systemischen Infektparametern gekennzeichnet, da die auslösenden Erreger häufig niedrigvirulente Erreger, wie z. B. Cutibacterium acnes oder Staphylococcus epidermidis sind.

**Table 2:** 

Jahr	Autor	Untersuchung	LoE	CoR	(*n* = x)	Sensitivität	Spezifität	Positive LR	Negative LR	Positiv prädiktiver Wert	Negativ prädiktiver Wert
2007	Bottner et al. [11]	–	I	I	78	–	–	–	–	–	–
		CRP (1,5 mg/dl)	–	–	–	0,95 (0,86–1,0)	0,91 (0,94–0,99)	10,86 (5,3–73,07)	0,05 (0,0–0,17)	0,8 (0,64–0,96)	0,98 (0,94–1,0)
		ESR (32 mm/h)	–	–	–	0,81 (0,64–0,98)	0,89 (0,82–0,97)	7,69 (3,47–38,2)	0,21 (0,02–0,44)	0,74 (0,56–0,92)	0,93 (0,8–1,0)
2004	Savarino et al. [71]	–	I	I	26	–	–	–	–	–	–
		ESR (50 mm/h)	–	–	–	0,6 (0,3–0,9)	0,94 (0,82–1,0)	9,6 (1,64–16,1)	0,43 (0,09–0,86)	0,86 (0,71–1,0)	0,79 (0,61–0,97)
		CRP (2 mg/dl)	–	–	–	0,38 (0,14–0,61)	0,7 (0,42–0,98)	1,25 (0,24–38,34)	0,89 (0,39–2,07)	0,67 (0,36–0,97)	0,42 (0,18–0,65)
1980	Kamme et al. [37]	–	I	I	63	–	–	–	–	–	–
		ESR (30 mm/h)	–	–	–	0,89 (0,8–0,99)	0,72 (0,54–0,9)	3,2 (1,75–9,54)	0,15 (0,01–0,37)	0,83 (0,71–0,94)	0,82 (0,66–0,98)
2007	Greidanus et al. [27]	–	I	I	151	–	–	–	–	–	–
		ESR (30 mm/h)	–	–	–	0,82 (0,71–0,93)	0,88 (0,81–0,94)	6,7 (3,84–15,52)	0,2 (0,07–0,36)	0,74 (0,62–0,86)	0,92 (0,87–0,97)
		CRP (1,0 mg/dl)	–	–	–	0,93 (0,86–1,0)	0,83 (0,76–0,9)	5,5 (3,57–10,23)	0,08 (0,01–0,18)	0,7 (0,58–0,82)	0,97 (0,93–1,0)
2007	Della Valle et al. [17]	–	I	I	94	–	–	–	–	–	–
		ESR (30 mm/h)	–	–	–	0,9 (0,81–0,99)	0,66 (0,53–0,79)	2,66 (1,74–4,68)	0,15 (0,01–0,35)	0,67 (0,55–0,8)	0,9 (0,8–0,99)
		CRP (1 mg/dl)	–	–	–	0,95 (0,89–1,00)	0,75 (0,64–0,87)	3,88 (2,45–7,86)	0,06 (0,02–0,18)	0,75 (0,63–0,87)	0,95 (0,89–1,0)
2008	Schinsky et al. [73]	–	I	I	201	–	–	–	–	–	–
		ESR (30 mm/h)	–	–	–	0,96 (0,91–1,0)	0,39 (0,31–0,47)	1,58 (1,33–1,91)	0,09 (0,0–0,28)	0,37 (0,29–0,45)	0,97 (0,92–1,0)
		CRP (1 mg/dl)	–	–	–	0,95 (0,89–1,0)	0,71 (0,94–1,0)	3,29 (2,45–4,69)	0,08 (0,0–0,18)	0,55 (0,45–0,65)	0,97 (0,94–1,0)
2007	Fink et al. [24]	–	I	I	145	–	–	–	–	–	–
		CRP (1,35 mg/dl)	–	–	–	0,73 (0,59–0,86)	0,81 (0,73–0,88)	3,81 (2,21–7,48)	0,34 (0,15–0,56)	0,59 (0,45–0,73)	0,89 (0,82–0,95)

*CRP* C‑reaktives Protein, *CoR* Class of Recommendation (EAST), *ESR* Erythrozytensedimentationsrate, *LoE* Level of Evidence (EAST), *EAST* Eastern Association for the Surgery of Trauma

### Fazit Laborparameter

Die Arbeitsgruppe sieht keine Möglichkeit zum sicheren Ausschluss bzw. der Bestätigung einer periprothetischen Infektion allein durch systemische Entzündungsparameter. Hierunter fallen auch neue moderne Entzündungsmarker wie Interleukine, Procalcitonin oder ähnliche. Die weitere Abklärung eines schmerzhaften Gelenkes sollte unabhängig von diesen Werten erfolgen.

## Fistel

Gemäß MSIS [76], IDSA [58], International Consensus Meeting [61] und EBJIS [68] beweist das Vorhandensein einer Fistel, die mit einer Prothese kommuniziert, die periprothetische Infektion [9, 80].

## Goldstandard Synoviaanalyse

Die Synoviaanalyse stellt den aktuellen Goldstandard in der präoperativen Diagnostik dar. Die aktuelle Literatur ist in Tab. 3 und 4 dargestellt. Obwohl die Komplikationsraten nach Gelenkpunktion von künstlichen Knie- und Hüftgelenken als niedrig einzuschätzen sind, müssen Patienten vor diesem Eingriff formal aufgeklärt werden (siehe auch Eingangskriterium).

**Table 3:** 

Jahr	Autor	Untersuchung	LoE	CoR	(*n*)	Sensitivität	Spezifität	Positive LR	Negative LR	Positiv prädiktiver Wert	Negativ prädiktiver Wert
2007	Della Valle et al. [17]	–	I	I	94	–	–	–	–	–	–
		Mikrobiologie	–	–	–	0,8	0,93	n.m.	n.m.	0,94	0,84
		Zellzahl (3,0 × 10^3^)	–	–	–	0,98 (0,93–1,9)	1	X	0,02	1	0,98 (0,94–1,0)
		Zelldiff.	–	–	–	0,98 (0,93–1,0)	0,85 (0,75–0,95)	6,46 (3,75–18,75)	0,03 (0–0,1)	0,83 (0,73–0,94)	0,98 (0,94–1,0)
2007	Fink et al. [24]	–	I	I	145	–	–	–	–	–	–
		Mikrobiologie	–	–	–	0,73 (0,59–0,86)	0,95 (0,91–0,99)	15,23 (6,64–125,4)	0,29 (0,14–0,45)	0,85 (0,73–0,97)	0,9 (0,85–0,96)
2008	Ghanem et al. [26]	–	I	I	429	–	–	–	–	–	–
		Zellzahl	–	–	–	0,91 (0,86–0,95)	0,88 (0,84–0,92)	7,59 (5,45–11,81)	0,11 (0,05–0,16)	0,82 (0,76–0,88)	0,94 (0,91–0,97)
		Zelldiff.	–	–	–	0,95 (0,92–0,98)	0,95 (0,92–0,97)	18,19 (11,62–38,43)	0,05 (0,02–0,09)	0,92 (0,87–0,96)	0,97 (0,95–0,99)
2007	Trampuz et al. [80]	–	I	I	–	–	–	–	–	–	–
		Zellzahl (1,7 × 10^3^)	–	–	–	0,94 (0,86–1,0)	0,88 (0,81–0,94)	7,76 (4,65–17,92)	0,07 (0,0–0,17)	0,73 (0,60–0,86)	0,98 (0,95–1,0)
		Zelldiff.	–	–	–	0,97 (0,91–1,0)	0,98 (0,95–1,0)	48,04 (19,07–136,76)	0,03 (0,0–0,09)	0,94 (0,87–1,0)	0,99 (0,97–1,0)
2012	Zmistowski et al. [88]	–	I	I	150	–	–	–	–	–	–
		Zellzahl (3,0 × 10^3^)	–	–	–	0,93 (0,87–0,99)	0,94 (0,88–0,99)	14,35 (7,28–99)	0,07 (0,01–0,14)	0,93 (0,87–0,99)	0,94 (0,88–0,99)
		Zelldiff.	–	–	–	0,93 (0,87–0,99)	0,83 (0,75–0,91)	5,52 (3,46–11,62)	0,08 (0,01–0,17)	0,84 (0,76–0,92)	0,93 (0,87–0,99)

*LoE* Level of Evidence (EAST), *CoR* Class of Recommendation (EAST), *n.m.* nicht möglich

**Table 4:** 

Jahr	Autor	Untersuchung	LoE	CoR	(*n* = x)	Sensitivität	Spezifität	Positive LR	Negative LR	Positiv prädiktiver Wert	Negativ prädiktiver Wert
1993	Barrack et al. [4]	–	I	I	291	–	–	–	–	–	–
		Mikrobiologie	–	–	–	0,6 (0,3–0,9)	0,88 (0,84–0,92)	5,11 (1,91–11,32)	0,45 (0,1–0,83)	0,15 (0,04–0,27)	0,98 (0,97–1,0)
1996	Mulcahy et al. [53]	–	I	I	71	–	–	–	–	–	–
		Mikrobiologie	–	–	–	0,69 (0,46–0,91)	0,91 (0,83–0,99)	7,56 (2,76–61,25)	0,34 (0,09–0,65)	0,69 (0,46–0,91)	0,91 (0,83–0,99)
2004	Williams et al. [84]	–	I	I	273	–	–	–	–	–	–
		Mikrobiologie	–	–	–	0,8 (0,71–0,9)	0,94 (0,9–0,97)	12,47 (7,23–29,34)	0,21 (0,11–0,32)	0,81 (0,72–0,91)	0,93 (0,90–0,97)
2004	Malhotra et al. [47]	–	I	I	41	–	–	–	–	–	–
		Mikrobiologie	–	–	–	0,44 (0,12–0,77)	0,91 (0,81–1,0)	4,74 (0,62–106,19)	0,61 (0,23–1,09)	0,57 (0,2–0,94)	0,85 (0,73–0,97)
2008	Schinsky et al. [73]	–	I	I	201	–	–	–	–	–	–
		Zellzahl (4,2 × 10^3^)	–	–	–	0,84 (0,74–0,93)	0,93 (0,89–0,97)	12,21 (6,75–33,94)	0,18 (0,07–0,29)	0,82 (0,72–0,92)	0,94 (0,90–0,98)
		Zelldiff. (80 %)	–	–	–	0,82 (0,72–0,92)	0,83 (0,77–0,89)	4,78 (3,08–8,36)	0,22 (0,09–0,37)	0,64 (0,53–0,76)	0,92 (0,88–0,97)
1999	Spangehl et al. [78]	–	I	I	183	–	–	–	–	–	–
		Zellzahl (5,0 × 10^3^)	–	–	–	0,36 (0,18–0,53)	0,99 (0,98–1,0)	55,36 (9,43–86,89)	0,65 (0,46–0,84)	0,91 (0,74–1,0)	0,9 (0,85–0,94)
		Zelldiff. (80 %)	–	–	–	0,89 (0,78–1,0)	0,85 (079–0,91)	5,94 (3,76–10,75)	0,13 (0,0–0,28)	0,52 (0,38–0,66)	0,98 (0,95–1,0)
2013	Dinneen et al. [19]	–	I	I	75	–	–	–	–	–	–
		Zellzahl (1,58 × 10^3^)	–	–	–	0,895 (0,783–0,997)	0,913 (0,827–0,999)	–	–	–	–
		Zelldiff. (65 %)	–	–	–	0,897 (0,795–0,999)	0,866 (0,761–0,971)	–	–	–	–

*LoE* Level of Evidence (EAST), *CoR* Class of Recommendation (EAST)

Die Analyse der Synoviaaspiration und die hieraus gewonnene mikrobiologische Kultur ist ein sehr kontrovers diskutiertes Thema in der Literatur. Die Kombination aus mikrobiologischer Langzeitbebrütung, Zellzahlbestimmung und Zelldifferenzierung entspricht dem aktuellen Goldstandard zur Analyse von Synoviaflüssigkeit. Die Zellzahl und Zelldifferenzierung sind äußerst wichtige Parameter der Synoviaanalyse. Einen entscheidenden Diskussionspunkt stellen die verwendeten Grenzwerte der Zellzahl und Zelldifferenzierung dar. Verschiedene Studien haben sich mit Grenzwertberechnungen von Zellzahl- und Zelldifferenzierungsuntersuchungen auseinandergesetzt. Trampuz et al. schlagen 1,7 × 10^3^/µl (Zellzahl) und 65 % (Anteil neutrophile Granulozyten, Zelldifferenzierung) vor, Zmistowski et al. und Della Valle beschreiben vergleichbare Ergebnisse, obwohl ein höherer Grenzwert von 3,0 × 10^3^/µl und 75 % gewählt wurde. Die Grenzwertbestimmung erfolgten mittels ROC(„receiver-operating characteristics“)-Kurven, diese Technik ist im Speziellen davon abhängig, welche Bakterien für die PPI in der ausgewählten Kohorte verantwortlich waren. Als Beispiel ist die Studie von Barrack et al. zu nennen, hier wurden als optimale Grenzwerte 4,2 × 103 µl bzw. 75 % verwendet. Das Kollektiv bestand aber überwiegend aus Patienten mit High-Grade-Infektionen und hochvirulenten Bakterien [73]. Die Expertengruppe hat sich aus diesem Grund auf einen niedrigeren Grenzwert im Algorithmus geeinigt, um auch chronische bzw. Low-Grade-Infektionen zu erfassen. Die Frage, ob Zellzahl und Zelldifferenzierung über den Grenzwerten liegen müssen, um von einer periprothetischen Infektion auszugehen, wurde ausführlich diskutiert. Als Konsens wurde festgelegt, dass entweder eine erhöhte Zellzahl oder eine erhöhte Zelldifferenzierung ausreicht, um eine Infektion wahrscheinlich zu machen.

Ein Problem der mikrobiologischen Kultur wird deutlich, wenn man die Literatur bezüglich der Sensitivitäten vergleicht. Hier zeigen sich sehr heterogene Daten: Der verursachende Erreger konnte in 44–80 % der Fälle durch eine mikrobiologische Kultur nachgewiesen werden [47, 84]. Ein entscheidender Faktor, der die Nachweiswahrscheinlichkeit deutlich erhöht, ist die Kulturdauer. Viele Studien mit geringer Sensitivität und Spezifität haben eine verkürzte Bebrütungszeit von 48 h angewendet bzw. die Kulturdauer nicht angegeben. Neben einer Bebrütungszeit von 14 Tagen ist die Antibiotikafreiheit bzw. das Absetzen einer bestehen Antibiotikatherapie mindestens 14 Tage vor Aspiration von Vorteil [70, 72]. Ein Problem sind falsch positive mikrobiologische Befunde, die oft durch Kontamination der mikrobiologischen Kultur verursacht werden. Dies führt, falls nicht durch einen Experten evaluiert und erkannt, zu einer massiven Übertherapie [86]. Hier ist es wichtig, dass die mikrobiologischen Resultate mit Nachweis von möglichen Kontaminanten (z. B. S. epidermidis, C. acnes) stets mit der Zellzahl im Punktat korreliert werden.

### Fazit Synoviaanalyse

Die Synoviaanalyse stellt den aktuellen Goldstandard dar. Als Grenzwerte empfehlen wir eine Leukozytenzahl von 1500/µl bzw. einen Granulozytenanteil von >65 %. Der Nachweis einer erhöhten Zellzahl *oder* einer erhöhten Zelldifferenzierung ist als Infektion zu werten. Eine Langzeitbebrütung der mikrobiologischen Kultur von 14 Tagen ist erforderlich. Bei Nachweis von potenziellen Kontaminanten muss stets die Zellzahl zur Interpretation herangezogen werden.

## Weitere Verfahren in der Synoviaanalyse

Andere neuartige synoviale Marker, wie der Alpha-Defensin-Test, zeigen vielversprechende Ergebnisse, jedoch sind die Fallzahlen in der aktuellen Literatur niedrig. Grundsätzlich muss zwischen einem kommerziell angebotenen Schnelltest (Minuten, qualitativ) und einem über verschiedene Labore angebotenen ELISA-Test (Tage, quantitativ) unterschieden werden. Die meisten Veröffentlichungen beziehen sich dabei auf den quantitativen ELISA-Test. Ein Problem stellen die selektiven Patientenkollektive dar, in vielen Studien wurden Patienten mit rheumatoider Arthritis oder abriebbedingter Metallose ausgeschlossen [14–16]. Kasparek et al. errechneten für den Alpha-Defensin-Test eine Sensitivität von 67 % bei einer Spezifität von 93 % [38]. Renz et al. errechneten eine Sensitivität von 84 % unter Verwendung der MSIS-Kriterien, von 67 % unter Verwendung der IDSA-Kriterien und 54 % unter Verwendung der PRO-IMPLANT/EBJIS-Kriterien [68]. Diese hochaktuellen Studien zeigten die noch bestehenden Probleme dieser neuartigen Biomarkertests. Konsens der Expertengruppe ist daher, dass die Alpha-Defensin-Bestimmung zum aktuellen Zeitpunkt nicht flächendeckend empfohlen werden kann.

Der Leukozytenesterasetest detektiert Leukozyten durch das in Leukozyten enthaltenen Enzym Leukozytenesterase. Durch eine chemische Reaktion kommt es am Teststreifen zu einem Farbumschlag, hierdurch wird semiquantitativ ein indirekter Leukozytennachweis im Punktat geführt. In einer Metaanalyse wird eine gepoolte Sensitivität von 81 % bei einer Spezifität von 97 % angegeben [85]. Aufgrund der problemlosen Verfügbarkeit und des extrem niedrigen Preises ist dieser Test als Konsens für eine Second-Line-Diagnostik unter bestimmten Umständen anwendbar. Insbesondere ist der Test aufgrund der hohen Spezifität bei positivem Resultat wegweisend. Wichtig ist hier die Limitation der fehlenden Verwertbarkeit bei Blutbeimengung des Punktats sowie die falsch positiven Resultate bei Nichteinhalten des Zeitfensters zur Ablesung des Resultates zu vermerken.

### Fazit neue Biomarker

Der flächendeckende Einsatz der Alpha-Defensin-Bestimmung kann bisher nicht empfohlen werden. Der Leukozytenesterasetest ist als Second-Line-Diagnostik verwendbar falls a) keine elektive Diagnostik erfolgt ist (z. B. aseptischer Wechsel mit intraoperativen Infektverdacht) oder b) trotz Algorithmus ein unklarer Befund vorliegt. Ein positiver Leukozytenesterasetest kann individuell als hinweisend in Betracht gezogen werden, schließt jedoch eine periprothetische Infektion weder mit ausreichender Sicherheit aus, noch bestätigt er diese.

## Biopsie von periprothetischem Gewebe zur mikrobiologischen und histologischen Aufarbeitung

Ein weiterer Schritt bei persistierendem Infektverdacht (z. B. beim Ausschluss anderer Pathologien, widersprüchlichen Ergebnissen in der Synoviaaspiration), welcher jedoch deutlich invasiver (und somit komplikationsreicher und kostenintensiver) ist, stellt die Synovialisbiopsie dar. Durch Entnahme von Biopsaten kann eine wie von Malhotra and Morgan in ihrem Kollektiv vorgeschlagene Kombination aus mikrobiologischer Aufarbeitung und histopathologischer Untersuchung durchgeführt werden. Die Autoren berichten von einer Sensitivität von 80 % bei einer Spezifität von 100 % durch eine synoviale Biopsie im Vergleich zu deutlich niedrigeren Werten bei der Synoviaaspiration. (44 %/91 %). Fink et al. zeigten an mikrobiologischen Biopsien deren Überlegenheit im Vergleich zur Kultur aus der Synovialflüssigkeit. In Ihrem Kollektiv erreichten sie Sensitivitäten von 100 % (K-TEP) und 87 % (H-TEP) bei einer Spezifität von 98 % [22, 23]. Pohlig et al. zeigten, wie die Sensitivität und Spezifität durch Kombination einer arthroskopisch gewonnen Biopsie mit mikrobiologischer und histopathologischer Untersuchung gesteigert werden können. Durch diese Kombination konnte die höchste Testgüte erreicht werden [64]. Als etabliertes Verfahren zur histopathologischen Einteilung ist die Morawietz-und-Krenn-Klassifikation zu nennen: hierbei werden die Granulozyten pro Gesichtsfeld in der periprothetischen Membran gezählt [40]. Die in den USA zum Teil etablierte Schnellschnittdiagnostik spielt in Deutschland eine eher untergeordnete Rolle. Im Gegensatz zur mikrobiologischen Biopsie spielt der Entnahmeort bei histopathologischen Proben eine außerordentlich wichtige Rolle. Ziel ist es, dass Teile der sogenannten SLIM („synovia-like interface membrane“) oder Neosynovialis prothesennah gewonnen werden [40]. Technisch gesehen erfolgt die Biopsie am Kniegelenk durch die Standardportale der Kniearthroskopie, am Hüftgelenk sind in leichter Hüftflexion ein hohes und ein tiefes anterolaterales Portal geeignet, wie sie üblicherweise zur Adressierung des peripheren Gelenkkompartiments verwendet werden. Es werden 6 Proben des periprothetischen Gewebes entnommen und davon 5 zur mikrobiologischen und eine zur histologischen Untersuchung eingeschickt [2, 49]. Trotz der hervorragenden Testgüte bei synovialen Biopsien (Tab. 5) sollte die Indikation streng gestellt werden. Ein persistierender Infektverdacht bei positiver Risikoanamnese, Frühlockerung oder persistierenden unklaren Infektparametern können dies rechtfertigen. Obwohl arthroskopische Operationen als kleine Eingriffe gelten, sind diese nicht risikolos. Potenzielle Risken sind neben der Verletzung von neurovaskulären Strukturen die postoperative Infektion sowie die Beschädigung von Prothesenkomponenten durch die Arthroskopieinstrumente. Zudem ist das Risiko eines „Sampling-Errors“ durch Nichterreichen des repräsentativen Gewebes und konsekutiven falsch negativen Ergebnissen zu berücksichtigen. Wie auch die Synoviaaspiration stellt die Indikation zur arthroskopischen Biopsie eine Risiko-Nutzen-Abwägung dar, in der ein maximaler diagnostischer Gewinn bei minimalem Patientenrisiko erzeugt werden soll.

**Table 5:** 

Jahr	Autor	Untersuchung	LoE	CoR	(*n* = x)	Sensitivität	Spezifität	Positive LR	Negative LR	Positiv prädiktiver Wert	Negativ prädiktiver Wert
2002	Banit et al. [3]	–	I	I	–	–	–	–	–	–	–
		Schnellschnitt (Hüfte)	–	–	63	0,45 (0,16–0,75)	0,92 (0,85–1,0)	5,91 (1,07–166,5)	0,59 (0,25–0,99)	0,56 (0,23–0,88)	0,89 (0,81–0,97)
		Schnellschnitt (Knie)	–	–	55	1	0,96 (0,9–1,0)	23,00 (9,76–64,7)	0	0,82 (0,59–1,0)	1
2007	Francés Borrego et al. [25]	–	I	I	–	–	–	–	–	–	–
		Schnellschnitt (Hüfte)	–	–	83	0,5 (0,15–0,85)	1	–	0,5 (0,15–0,85)	1	0,95 (0,9–1,0)
		Schnellschnitt (Knie)	–	–	63	0,67 (0,48–0,86)	0,9 (0,8–0,99)	6,5 (2,42–116,4)	0,37 (0,15–0,65)	0,8 (0,62–0,98)	0,81 (0,7–0,93)
2008	Schinsky et al. [73]	–	I	I	201	–	–	–	–	–	–
		Schnellschnitt (Hüfte)	–	–	–	0,73 (0,61–0,84)	0,94 (0,9–0,98)	11,8 (6,06–37,34)	0,29 (0,16–0,43)	0,82 (0,71–0,92)	0,9 (0,85–0,95)
2008	Fink et al. [23]	–	I	I	145	–	–	–	–	–	–
		Fixation	–	–	–	1	0,98 (0,95–1,0)	52,5 (22,13–140,88)	X	0,95 (0,89–1,0)	1
2007	Della Valle et al. [17]	–	I	I	94	–	–	–	–	–	–
		Schnellschnitt (Knie)	–	–	–	0,88 (0,78–0,98)	0,96 (0,91–1,0)	23,27 (8,74–72,1)	0,13 (0,02–0,24)	0,95 (0,88–1,0)	0,91 (0,84–0,99)
2013	Fink et al. [22]	–	I	I	100	–	–	–	–	–	–
		Fixation (Knie)	–	–	–	0,62 (0,48–0,76)	1	–	0,38 (0,24–0,52)	1	0,76 (0,67–0,86)

*LoE* Level of Evidence (EAST), *CoR* Class of Recommendation (EAST)

### Fazit Biopsie

Bei Diskrepanz zwischen Infektverdacht und unklaren Punktionsergebnissen sollte nach Ausschluss anderer Pathologien periprothetisches Gewebe gewonnen werden. Durch Kombination von mikrobiologischer bzw. histopathologischer Untersuchung der Biopsien kann die höchste diagnostische Güte erreicht werden – vorausgesetzt die Proben sind repräsentativ.

Die präoperativ vorliegenden Resultate müssen stets mit den im Rahmen der Revisionsoperation entnommenen Proben korreliert und die Diagnose nach Vorliegen dieser erneut kritisch analysiert werden. Die intraoperativ entnommenen Biopsien sind oft aussagekräftiger, da das repräsentative Gewebe (z. B. Interface zwischen Prothese und Knochen) erreicht wird. Zudem kann die explantierte Prothese zur Sonikation geschickt werden, welche sich vor allem bei Low-Grade-Infektionen als sensitiver als Gewebeuntersuchungen erwies. Eine negative präoperative Diagnostik verringert die Wahrscheinlichkeit einer Infektion beträchtlich, schließt sie jedoch nicht gänzlich aus. Analog muss ein positiver präoperativer Test (z. B. mikrobiologischer Nachweis eines typischen Kontaminanten oder eine leicht erhöhte Zellzahl) immer mit der intraoperativen Diagnostik korreliert werden, um eine Überdiagnose zu vermeiden.

## Fazit für die Praxis

- Der Ausschluss einer periprothetischen Infektion ist von enormer Bedeutung vor einer Revisionsoperation und entscheidet in vielen Fällen über den Erfolg und die Invasivität der Operation.
- Die Diagnose „periprothetische Infektion“ erfordert eine substanzielle Veränderung der therapeutischen Strategie.
- Der durch die Arbeitsgruppe entwickelte Algorithmus fasst Positionen aus der aktuellen Literatur und spezielle Expertenmeinungen zusammen.
- Da die Weiterentwicklung der Diagnostik ein dynamischer Prozess ist, erfolgt die jährlich Reevaluation. Dies ermöglicht einen transparenten diagnostischen Ansatz im Sinne einer Standard-Operation-Procedure.
- Hierdurch wird unabhängig von der Erfahrung des Untersuchers eine hohe Prozessqualität erreicht.

## Abkürzungen

AAOS: American Academy of Orthopaedic Surgeons
AMP: Antimikrobielle Peptide
BSG: Blutsenkungsgeschwindigkeit
CoR: Class of Recommendation
CRP: C‑reaktives Protein
EBJIS: European Bone and Joint Infection Society
ELISA: Enzyme-linked Immunosorbent Assay
ESR: Erythrozytensedimentationsrate
H‑TEP: Hüfttotalendoprothese
IDSA: Infectious Diseases Society of America
ISO: International Organization for Standardisation
ITU: International Telecommunication Union
K‑TEP: Knietotalendoprothese
LoE: Level of Evidence
MSIS: Musculoskeletal Infection Society
PCR: Polymerase-Kettenreaktion
PPI: Periprothetische Infektion
ROC: „Receiver-operating characteristics“
SIRS: Systemisches inflammatorisches Response-Syndrom
SLIM: „Synovia-like interface membrane“
SOFA: Sequential Organ Failure Assessment
